# Assessed and discharged – diagnosis, mortality and revisits in short-term emergency department contacts

**DOI:** 10.1186/s12913-022-08203-y

**Published:** 2022-06-23

**Authors:** Hassan Al-Mashat, Tim A. Lindskou, Jørn M. Møller, Marc Ludwig, Erika F. Christensen, Morten B. Søvsø

**Affiliations:** 1 Centre for Prehospital and Emergency Research, Aalborg University and Aalborg University Hospital, Sdr. Skovvej 15, Aalborg, Denmark; 2 Emergency Department & Trauma Centre, Aalborg University Hospital, Aalborg, Denmark; 3 Emergency Department Hjørring, North Denmark Regional Hospital, Hjørring, Denmark

**Keywords:** Hospital Emergency Service, Mortality, Length of stay

## Abstract

**Background:**

Emergency departments (EDs) experience an increasing number of patients. High patient flow are incentives for short duration of ED stay which may pose a challenge for patient diagnostics and care implying risk of ED revisits or increased mortality. Four hours are often used as a target time to decide whether to admit or discharge a patient.

**Objective:**

To investigate and compare the diagnostic pattern, risk of revisits and short-term mortality for ED patients with a length of stay of less than 4 h (visits) with 4–24 h stay (short stay visits).

**Methods:**

Population-based cohort study of patients contacting three EDs in the North Denmark Region during 2014–2016, excluding injured patients. Main diagnoses, number of revisits within 72 h of the initial contact and mortality were outcomes. Data on age, sex, mortality, time of admission and ICD-10 diagnostic chapter were obtained from the Danish Civil Registration System and the regional patient administrative system. Descriptive statistics were applied and Kaplan Meier mortality estimates with 95% CI were calculated.

**Results:**

Seventy-nine thousand three hundred forty-one short-term ED contacts were included, visits constituted 60%. Non-specific diagnoses (i.e. *symptoms and signs* and *other factors*) were the most frequent diagnoses among both visits and short stay visits groups (67% vs 49%). Revisits were more frequent for visits compared to short stay visits (5.8% vs 4.2%). *Circulatory diseases* displayed the highest 0–48-h mortality within the visits and *infections* in the short stay visits (11.8% (95%CI: 10.4–13.5) and (3.5% (95%CI: 2.6–4.7)). 30-day mortality were 1.3% (95%CI: 1.2–1.5) for visits and 1.8% (95%CI: 1.7–2.0) for short stay visits. The 30-day mortality of the ED revisits with an initial visit was 1.0% (0.8–1.3), vs 0.7% (0.7–0.8) for no revisits, while 30-day mortality nearly doubled for ED revisits with an initial short stay visit (2.5% (1.9–3.2)).

**Conclusions:**

Most patients were within the visit group. Non-specific diagnoses constituted the majority of diagnoses given. Mortality was higher among patients with short stay visits but increased for both groups with ED revisits. This suggest that diagnostics are challenged by short time targets.

## Background

The role of emergency departments (EDs) is of importance both for the patients and the health care system. It does not only provide around the clock treatment for the acutely ill patients, but also provides the first steps in evaluating and investigating patients for diseases. This will often result in an out- or in-patient referral to the different specialized departments in the hospital.

Internationally, the EDs are confronted with a growing demand for their services as the number of unplanned contacts increases [1–4].

Handling many contacts requires a high flow environment where patients are assessed within few hours of their arrival and a decision is made – is there a need for further observation, investigations, admission or can the patient be discharged? In this process, assigning a diagnosis to the patient within a short time frame is important for initiating treatment and further investigations. However, if ED-stay is too short it might result in incomplete or fragmented patient diagnostics, as physician medical decision making may also be abbreviated under shorter ED visits, which in turn could lead to increased risk of ED revisits or increased short-term mortality.

In the United Kingdom, the increased ED waiting times and increased demand in the National Health Services, was addressed in 2000 where the performance standard of “a maximum of four-hour wait from arrival to admission, transfer or discharge” was introduced for all patients [5]. The performance standard was later revised to at least 95% of all patients [6].

The current form of EDs in Denmark is relatively new since all EDs were centralized (40 hospitals were reorganized into 21 larger hospitals in 2007–10, and acute patient intake were aimed at EDs in the larger hospitals) and emergency medicine as a specialty was approved in 2018.

EDs are the common entrance for all trauma and acute medical conditions in Denmark. Certain conditions may bypass the ED to go to highly specialized department (e.g., stroke, myocardial infarction, and births). The EDs have a unit for patients expected to be discharged after a few days of treatment. Access to the ED is only possible by contacting the general practitioner (daytime and out-of-hours), or the Emergency Medical Services. If follow-up or additional care is needed, this is done by the primary care provider (general practitioner) or at certain outpatient clinics not affiliated with the ED although the ED also has a very small outpatient function. Danish EDs have no uniform performance standards for time, but several hospitals use a four hour target for time-to- decision or similar [7]. A nationwide Danish study investigated the discharge diagnose pattern in the country’s EDs between 2005 to 2016. The proportion of patients diagnosed with injuries varied from 27.5 to 35.6%

These trauma patients often have short term stays that are quickly resolved, whereas patients with medical illness is a more diverse group.

By using the unique high-quality Danish health registers which also includes length of stay, it is possible to explore ED patients diagnoses and outcomes.

The aim of this study is to investigate and compare the diagnostic pattern of non-trauma patients, risk of revisit and short-term mortality for ED patients with a short ED stay, of 0–4 h, compared to 4–24 h. We hypothesize that those with a short length of stay will have higher rate of mortality and revisits than those with longer length of stay.

## Methods

### Study design

A population-based cohort study of patients contacting EDs in the North Denmark Region during January 2014 – December 2016.

The study is reported according to STrengthening the Reporting of OBservational studies in Epidemiology (STROBE) guidelines [8].

### Setting

The North Denmark Region is inhabited by approximately 587,000 people [9] all of which are provided with free, around-the-clock healthcare.

Every Danish citizen has a unique personal identification number (PIN), which is registered at hospital contacts [10]. Hospitals diagnose all patient contacts according to the International Classification of Disease-10 (ICD-10) system [11].

### Study population

All patients who had a non-trauma contact with one of the three somatic EDs (one of which is a university hospital) in the North Denmark Region during January 1^st^ 2014 to December 31^st^ 2016 were included. We excluded the trauma patients, who often have short quickly resolved medical issues, to focus on the patients with medical illness.

Patients not residing in the North or Central Denmark Region were excluded as follow-up was not possible for these patients. Furthermore, patients registered as ED patients, although transferred directly to specialized ward, were excluded. Patients registered as dead upon arrival at the ED were also omitted from the study and patients with ED contact durations longer than 24 h were excluded.

### Included variables

Time of admission, and ICD-10 diagnoses were retrieved from the regional patient administrative system. Data on age, sex and vital status, i.e. mortality, from the Danish Civil Registration System were obtained [10].

### Outcomes

Study outcomes were defined as; number of short-term patients at the ED and number of revisits within 72 h, distribution of diagnostic main chapters and lastly, 0–48 h- and 30-day mortality. For patients with revisits, 30-day mortality was assessed from their initial ED contact.

### Definitions

We defined two patient groups: those with a visit at the EDs with a duration of 0–4 h; from heron referred to as visits, and those with a visit or admittance with a duration of 4–24 h; referred to as short stay visits.

Individual patients may have had several visits or short stay visits in the study period.

Revisits was defined as an additional contact with the EDs within 72 h of the initial contact. Only the revisit immediately following the initial contact was used, although patients may have had several contacts within the 72 h. The limit of 72 h was decided to include patients whose initial medical issue may not have been resolved at the initial ED visit or short stay visit. Patients with a revisit later than 72 h were assessed as having greater possibility of presenting with a new medical issue.

### Statistical analyses

All patient data were anonymized prior to analysis. The results are presented as descriptive statistics with percentages of the diagnoses- and sex distribution, as well as median age.

In the reporting of diagnosis distribution, values less than 5 were omitted in order not to report patient micro data. Two-sample test of proportion was used to compare the two short-term ED contact groups. Kaplan–Meier estimator, with 95% confidence intervals (CI) was used to assess mortality. Date of death was also obtained for dates later than the study period, allowing follow-up for all patients to be included.

All statistical analyses were carried out with STATA 15.1 (Texas, USA).

## Results

### Diagnostic pattern

**Fig. 1:**
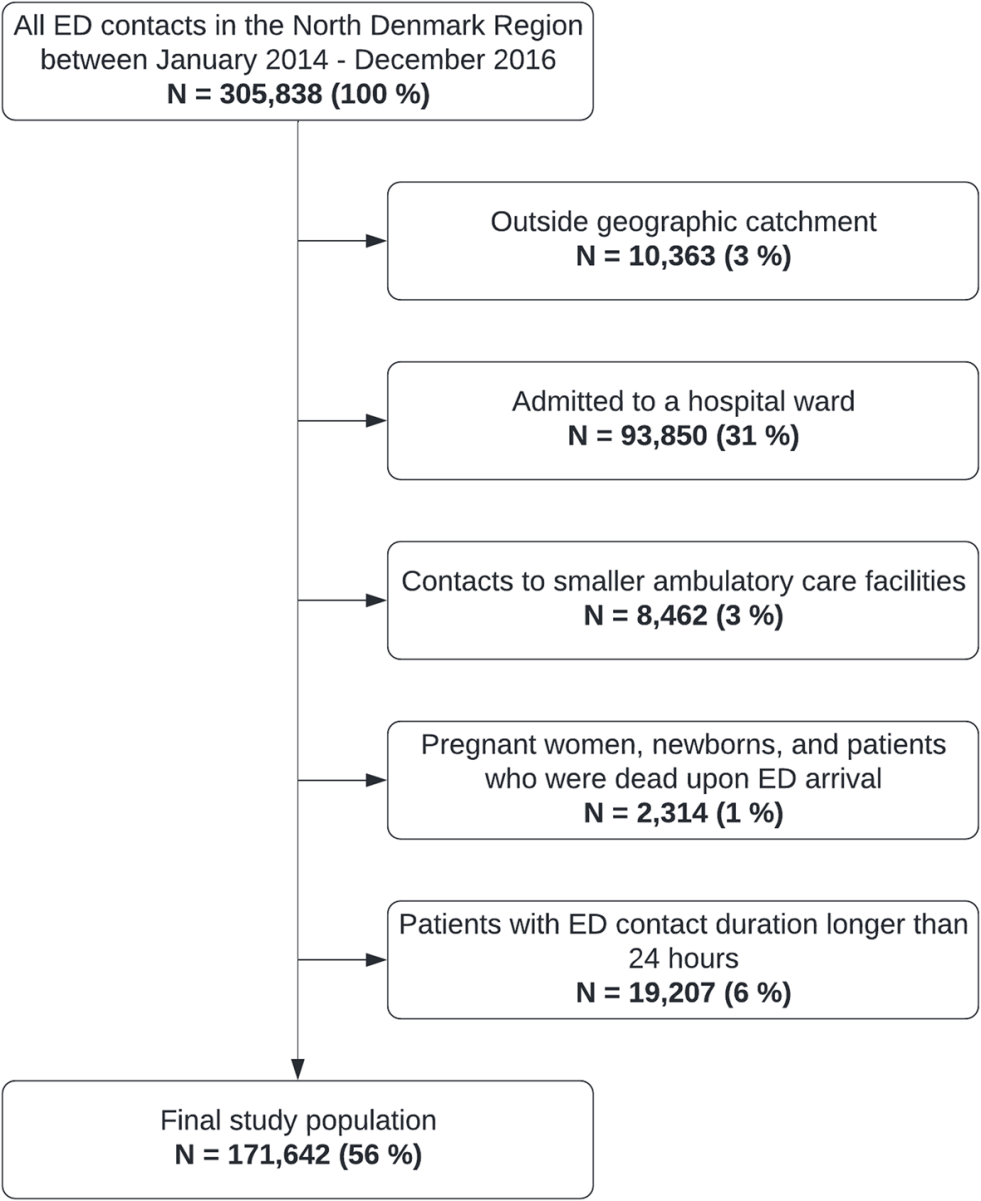
Flow chart of study population inclusion process

There was a total of 171,642 ED contacts (Fig. 1), and of these, 92,301 (54%) were diagnosed as trauma patients*,* who were not included.

In total, the study included 79,341 patients, of which, visits amounted 60% of these (47,809 visits vs 31,532 short stay visits).

In visits, patients received diagnoses from the two ICD-10 chapters other factors and symptoms and signs in 67% of all cases. This was followed by musculoskeletal (6.6%), respiratory (4.4%) and circulatory diseases (3.6%).

Symptoms and signs and other factors were also the most frequent diagnostic chapters used among short stay visits patients, constituting 49% of all cases. *Digestive diseases* contributed with 10.8%, whereas diagnoses concerning respiratory and musculoskeletal diseases were assigned in 7.0% and 5.4% of contacts, respectively. Details on the distribution of diagnoses are shown in Table 1.

**Table 1:** Characteristics and diagnostic pattern

Visits (n 47,809)			Short stay visits (n 31,532)		
**ED Contact**	**Overall**	**Revisit**	**ED Contact**	**Overall**	**Revisit**
**Age (median, range)**	39 (0–104)	*35 (0–100)*	**Age (median, range)**	48 (0–104)	*44 (0–100)*
**Sex (% female)**	49.5	*44.27*	**Sex (% female)**	50.6	*47.66*
**Length of stay (mean)**	1 h 52 m		**Length of stay (mean)**	11 h 27 m	
**ICD-10 main chapter (n, %)**			**ICD-10 main chapter (n, %)**		
Other factors	18,418 (38.5)	1300 (47.0)	Symptoms and signs	9481 (30.1)	324 (25.3)
Symptoms and signs	13,645 (28.5)	558 (20.2)	Other factors	5955 (18.9)	240 (18.8)
Musculoskeletal diseases	3157 (6.6)	206 (7.4)	Digestive diseases	3370 (10.7)	102 (8.0)
Respiratory diseases	2113 (4.4)	151 (5.5)	Respiratory diseases	2212 (7.0)	97 (7.6)
Circulatory diseases	1710 (3.6)	74 (2.7)	Musculoskeletal diseases	1717 (5.4)	84 (6.6)
Mental disorders	1660 (3.5)	72 (2.6)	Circulatory diseases	1658 (5.3)	71 (5.5)
Digestive diseases	1562 (3.3)	74 (2.7)	Mental disorders	1330 (4.2)	104 (8.1)
Skin diseases	1468 (3.1)	111 (4.0)	Genitourinary diseases	1228 (3.9)	47 (3.7)
Infections	1145 (2.4)	92 (3.3)	Infections	1192 (3.8)	42 (3.3)
Genitourinary diseases	874 (1.8)	45 (1.6)	Skin diseases	871 (2.8)	59 (4.6)
Remaining chapters	1982 (4.1)	84 (3.0)	Remaining chapters	2518 (8.0)	110 (8.6)
**Total**	**47,809 (100)**	**2767 (100)**	**Total**	**31,532 (100)**	**1280 (100)**

Characteristics and diagnostic pattern of patients with a visit (duration of 0-4 h) and short stay visits (duration of 4-24 h), Furthermore, the number of patients with a revisit within 72 h

### ED revisits

Overall, more visits patients had a revisit within 72 h than the short stay visits patients, 5.8% vs 4.2%, (*p* < 0.00). ICD-10 main chapter distribution largely followed the same pattern as the overall distribution, i.e. patients initially diagnosed with symptoms and signs (20% among visits and 25% among short stay visits) and other factors (47% and 19%) also appeared most frequently among revisits. (Table 1).

### Mortality

Patients with visits had an overall 0–48-h mortality of 0.7% (CI: 0.7–0.8), (*N* = 353) and the highest mortality rates were observed for circulatory diseases (11.8% (CI: 10.4–13.5), *N* = 204) and respiratory diseases (2.0% (CI: 1.5–2.7), *N* = 42).

Among patients with short stay visits, the overall 0–48-h mortality was 0.9% (CI: 0.8–1.0) (*N* = 277) and the diagnostic chapters with the highest mortality rates were infections (3.5% (CI: 2.6–4.7), *N* = 42), circulatory (3.4% (CI: 2.7–4.4), *N* = 57) and respiratory diseases (2.8% (CI: 2.2–3.6), *N* = 63).

The overall 30-day mortality rate was 1.3% (CI: 1.2–1.5) for visits patients(*n* = 640) of which most deaths were attributed to circulatory diseases (*N* = 224) and non-specific diagnoses; other factors (*N* = 158), symptoms and signs (*N* = 117). For short stay visits patients, the overall mortality was 1.8% (CI: 1.7–2.0) (*N* = 578) and the deaths were more evenly distributed between several chapters. (Table 2).

**Table 2:** 30-day mortality rate for visits and short stay visits patients

	**Visits patients**		**Short stay visits patients**	
**ICD-10 main chapter**	**Deaths (n)**	**Mortality rate**	**Deaths (n)**	**Mortality rate**
Overall	640	1.3 (1.2–1.5)	578	1.8 (1.7–2.0)
Symptoms and signs	117	0.8 (0.7–1.0)	107	1.1 (0.9–1.4)
Circulatory diseases	224	13.0 (11.5–14.7)	82	5.0 (4.0–6.1)
Respiratory diseases	51	2.4 (1.8–3.1)	81	3.6 (2.9–4.5)
Other factors	158	0.8 (0.7–1.0)	76	1.3 (1.0–1.6)
Digestive diseases	11	0.7 (0.3–1.3)	63	1.9 (1.5–2.4)
Infections	13	1.1 (0.6–2.0)	47	4.0 (3.0–5.2)
Blood diseases	9	4.0 (2.1–7.5)	32	6.3 (4.5–8.8)
Endocrine diseases	17	3.3 (2.0–5.2)	25	3.2 (2.1–4.6)
Genitourinary diseases	6	0.7 (0.3–1.5)	22	1.8 (1.2–2.7)
Remaining chapters	34	0.4 (0.3–0.6)	43	0.9 (0.6–1.1)

Cumulative number of deaths at day 30 for visit (duration of 0-4 h) and short stay visits (duration of 4-24 h) patients and corresponding Kaplan–Meier estimator mortality rates with 95% confidence intervals

Overall, 30-day mortality among visits patients who had a revisit within 72 h, were 1.0% (0.8–1.3), (*N* = 46), in contrast to those with no revisit 0.7% (CI: 0.7–0.8), (*N* = 594). *Symptoms and signs* displayed a higher mortality rate when patients had a revisit; 0.8% (CI: 0.7–1.0) (*N* = 105) vs 2.2% (CI: 1.2–3.8) (*N* = 12). On the contrary, patients with no revisit following an initial visit with *circulatory disease,* had a higher mortality rate (13.3% (CI: 11.7–15.0), (*N* = 219)) than those with revisits (6.8% (CI: 2.9–15.5), (*N* = 5)).

For short stay visits patients with a revisit within 72 h, the overall 30-day mortality rate doubled (2.5% (CI: 1.9–3.2), *N* = 43, compared to 1.4% (CI: 1.3–1.5), *N* = 535). Especially, *circulatory diseases* doubled in mortality rate among patients with revisits compared to patients without (10.0% (CI: 4.9–19.8), *N* = 7) vs. 4.7% (CI: 3.8–5.9), *N* = 75).

## Discussion

In this study of non-trauma ED contacts, visits patients comprised the majority, and the most prominent diagnoses were non-specific, i.e. other factors and symptoms and signs*,* meaning that the patients were assigned symptom-based diagnoses not organ or etiology based, specific diagnoses. With increased visit duration, however, more patients received organ-and etiology specific diagnoses. Primarily reporting on non-trauma patients was chosen as a high prevalence of injuries is to be expected in the ED, especially among short-term contacts [12, 13].

Non-specific diagnoses are often used when no specific organ-related disease is found. In line with this, a nationwide American study of the three most common ED complaints found that many patients did not receive a pathological discharge diagnosis (symptom-based rather than disease-specific diagnoses, i.e., abdominal pain versus biliary colic). The most common complaints were chest-pain, abdominal pain or headache and in 2009 pathological discharge diagnoses were given in 52%, 66% and 70% of these cases, respectively. During the study period, the proportion without pathological discharge diagnoses increased and since the three complaints constituted two-thirds of the discharges, which may suggest that the high number of non-specific diagnoses are experienced in other countries [14]. Furthermore, the study discusses that some doctors argue that obtaining a definitive, pathological diagnosis is often not possible in the ED setting and the goal in the ED should be to “rule out” life-threatening diseases and not to make pathological diagnoses.

Furthermore, a nationwide Danish study likewise found an increasing proportion of non-specific diagnoses in the period 2005 to 2016, with symptoms and signs and other factors contributing to 25.4% of all discharge diagnoses [1].

This current study demonstrated that the proportion of revisits was slightly higher for visits compared to short stay visits. For all revisits, overall mortality was also slightly higher. This could imply that a short-term contact, especially 4 h or less may in some cases not be sufficient to observe and diagnose a patient. The higher proportion of non-specific diagnoses among visits patients compared to short stay visits patients support this. On the other hand, mortality for the visits patient group as a whole was low and lower than in the short stay visits patients, perhaps indicating a low degree of severity among most patients in this group.

A Belgian study investigated patients who returned to the emergency department within 72 h following discharge from the ED [15]. The study reported a 72-h return rate of 2.2%. Likewise an American study demonstrated a 2.6% return rate within 7-days [16]. These rates contrast the current study’s 5.8% and 4.2% for visits and short stay visits respectively. This difference could be explained by the Belgian ED only took care of patients above 16 years of age, and the American above 18, whereas the current study included all ages.

The same Belgian and American studies reported that the majority of the revisits was related to the diagnosis given to the patients in the initial contact [15, 16]. This is in accordance with what this study has found. However, the American study found that the most common ED discharge diagnoses associated with a revisit (bounce back admission) were *‘chronic renal disease not end stage’*, *‘end stage renal disease’* and ‘*congestive heart failure’ *[16]. The ED setups throughout the world are likely not similar. This, in combination with ICD-10 diagnoses reported at chapter level only, as well as the use of all contacts to the ED not restricted to admissions only, may explain the differences in results.

Revisits may not only be due to patient deterioration. A previous study has indicated the most common reason behind why patients return to the ED is primarily uncertainty and fear regarding their medical condition, and having a diagnosis matters greatly for the patients [17]. Absence of trust in the healthcare system in attending the patients’ needs was reported as another reason [18].

### Limitations

A limitation with this study was that patients may have had several contacts within 72 h which were not accounted for in this study, as only the revisit immediately after the first contact was used, meaning that the number of revisits may have been underestimated, however our estimations indicate that less than 1% of the patients had more than one revisit, which is unlikely to have affected our results.

Furthermore, this study did not investigate the specific diagnoses of patients but rather diagnostic chapters.

The North Denmark Region constitute approximately 10% of the Danish population, and the population is in general older compared to other regions. However, the overall uniformity of the Danish ED’s system aids the external validity in Denmark and certain European nations, but may lack in external validity the rest of the world.

### Conclusion

Non-specific diagnoses were dominant among ED patients within the first 24 h and especially frequent, constituting two-thirds among those with the shortest stays, meaning they were discharged without any organ-or cause specific diagnosis. One out of 20 patients with the shortest stays had an ED revisit within 72 h, especially among those with non-specific diagnoses, and mortality was higher among patients with revisits. All together this indicates that diagnostics may be challenged by short time targets. We consider these findings a relevant input to current debate on the organization of EDs, which may interest health care planners.

## Data Availability

The data that support the findings of this study are available from the North Denmark Region but restrictions apply to the availability of these data, which were used under license from the Danish Data Protection Agency for the current study, and so are not publicly available. However, if approval from the Danish Data Protection Agency is obtained, data is available from the Emergency Medical Services in the North Denmark Region upon request.

## Abbreviations

ED: Emergency departments
PIN: Personal identification number
ICD-10: International Statistical Classification of Diseases, 10th Edition
CI: Confidence interval
